# Rupture of an Ovarian Cyst by a Fall

**Published:** 1845-12-01

**Authors:** James P. White


### Case of Ovarian Cyst, ruptured by a fall,

*Effusion of its contents into Peritoneal cavity, absorption, and permanent cure.*

Communicated for the Buffalo Medical Journal by JAMES P. WHITE, M. D.

A widow lady, 43 years of age, discovered during pregnancy, nearly 15 years since, a tumour in the left side about the size of an egg. She was safely delivered of a healthy child at the usual period, and has since given birth to three vigorous children, the youngest being now eight years old. The husband died before the birth of the last child. After the last confinement the tumor increased with considerable rapidity, and gave rise to much uneasiness. She had several attacks of what her physicians called inflammation of the bowels, and was treated by bleeding, blistering, cathartics, &c. The tumor has been during the last few years seen by Drs. Colgrove, B. Burwell, Bissell, Congar, and others, most, or all of whom diagnosed ovarian disease, and did not predict a favorable termination. I incidentally saw and examined the patient last summer, and found the left ovary larger than the head of a full grown fœtus, extending quite across the umbilical, into the right iliac region. Did not recommend the patient to institute any treatment for permanent relief.

In February last, on making a sudden effort on an icy stone step, she slipped, and fell with her whole weight upon the abdominal tumor. The shock was so severe as to produce prostration and syncope, and a messenger was despatched for me. After administering a cordial I proceeded to make an examination, and found the circumscribed character of the tumor entirely lost. There was a general fluctuation of the whole abdomen, and the peritoneum seemed the receptacle of a fluid escaped from the ruptured sac. The injury was followed by severe peritonitis, which yielded to bleeding, general and topical, with the usual adjuvants. The patient passed more than the usual quantity of high colored urine, and vomited frequently throughout her illness. After a gradual convalescence she seems now entirely restored, and in better health than she has enjoyed for several years. The strictest examination did not, during a visit last evening made for that purpose, enable me to detect the least vestige of the old tumor in the ovarian region. The menses have been, and are now regularly secreted, the respiration is full and complete, and she can lay upon either side without difficulty, which she has been unable to do until recently.

On reviewing the circumstances in the history of the foregoing case, several questions of pathological and practical importance, are very naturally suggested. Was this tumor really an ovarian cyst, filled with some fluid or soft matter? If so, was it ruptured by the force of the blow at the time of the fall? Was the peritonitis which followed, and during the continuance of which the fluid was probably absorbed, occasioned by the injury; or the result of the irritation produced by the sudden introduction into a serous membrane of a fluid more or less stimulating? Or, what is perhaps most probable, was it the combined effect of both? Granting the answers to these questions which seem most probable, can any deductions of practical importance be fairly drawn therefrom? Does the favorable termination of this case afford any encouragement for a resort to any of the operations for the radical cure of encysted dropsy, recommended by some modern practitioners? It is remarked by Dr. Sympson, in a "treatise on Dropsy of the Ovary," that it has been suggested to open the sac and discharge its contents into the peritonal cavity. But, so far as I am aware, the practice has never been resorted to. This operation would be very analogous to what probably resulted from the injury in the case related. The operations for extirpation, and the insertion of the seton, or the tube as recommended by Dr. Trowbridge, have been had recourse to sufficiently to dispel the great apprehension of danger which has prevailed in relation to opening the abdominal cavity. That suggestions may be made in relation to the history of the above case by those who saw it; or its practical application and usefulness is my only object in bringing it before the society. It will not be denied that the subject demands investigation, for notwithstanding the great variety of operations resorted to, and with partial success, still none is as yet adopted by the profession at large.—Indeed we may safely subscribe to the observations of Dr. Hunter, published half a century ago, "that though he had seen many cases of dropsy of the ovary treated by physicians of the first rank, he had never seen one cured. That in the present state of medical science it is an incurabte disease, and that a patient will have the best chance of living longest under it, who does least to get rid of it."

NOTE BY THE EDITOR.

The preceeding case was reported to the Buffalo Medical Association in September last, and would have appeared in the last number of the Journal, but for the accumulation of matter previously received. It is certainly an interesting case, and the practical questions which it suggests are of great importance. With reference to the latter, it is desirable to determine what were the circumstances directly involved in the cure. Any therapeutical deductions, will, obviously, hinge upon this point. The mere discharge of the contents of the cyst, was probably not in itself sufficient. If it were, it would have answered equally well if they had been evacuated through the abdominal walls by means of the trochar. Did the fact of the effusion into the peritoneal cavity, and the consecutive peritonitis, play an important part in the cure? We are unable to perceive why it should be so: and if not, the case affords no sanction for discharging the contents of ovarian tumours into this cavity by subcutaneous incisions, as has been proposed. The case, it is true, presents an illustration of the fact that the peritoneum is capable of absorbing the contents of an ovarian tumour, but if the peritoneal inflammation, thereby induced, exerts reciprocally no curative effect upon the ovarian cyst, it follows that there is nothing gained by imposing the duty of absorption upon the peritoneum, and exposing the patient to the dangers incident to peritonitis. We suppose the true explanation to be, that the force of the blow received upon the abdomen, which caused a rupture of the cyst, gave rise to a certain amount and kind of inflammatory action *in the cyst*, which ultimated in a radical cure on the same principle that hydrocele is cured by stimulating injections, or other means to develope inflammation in the tunica vaginalis. Indeed, cases of radical cure of hydrocele from bursting the tumour by an accidental blow are on record. If this be the true rationale, the evacuation of the fluid into the peritoneal cavity, and the consecutive peritonitis, have no direct, causative relation to the fortunate result; and it would certainly be unwise to imitate the case in these particulars by any surgical operation. With this view of the method of cure, the case, in so far as it admits of practical deductions, goes to sustain the practice which some surgeons have pursued, of evacuating the contents of the cyst by tapping, and then, by injections, the introduction of a foreign substance, or other means, exciting a certain degree of inflammation within the cyst—in short, applying to these cases the same principles of treatment which are employed in hydrocele. Of the merits of this plan we are not prepared to speak. Dr. Trowbridge's method of introducing tubes, to which Dr. White refers, is described by himself in a communication made to the Boston Medical and Surgical Journal, August, 1841.

In the American Journal of Medical Sciences, for October, 1845, our readers will find an article excerpted from the London and Edinburg Medical Journal, detailing a case in which, in the same individual, a spontaneous rupture of an ovarian cyst took place, with an escape of its contents into the peritoneal cavity, and subsequent absorption, three separate times. Peritonitis, on each occasion, was developed in this case, which terminated favorably; not, however, it will be observed, in a radical cure of the ovarian dropsy. On the contrary, the accumulation returned a second, and third time, notwithstanding the peritonitis. The ultimate issue of the case is not given. This case goes to confirm the opinion which we have expressed concerning the rationale of the case reported by our correspondent.

